# Abstract of the Proceedings of the Buffalo Medical Association, March 7th, 1876

**Published:** 1876-05

**Authors:** 


ART. IV.—*Abstract of the Proceedings of the Buffalo Medical Association.* Tuesday evening, March 7, 1876.

The Vice-President, Dr. Wyckoff, in the chair. Present—Drs. Cronyn, Howe Mynter, Samo, O'Brien, Lothrop, Bartlett, Briggs, Hopkins and Brush. By invitation, Dr. Baker. Minutes read and approved. Applications for membership were received from Drs. S. S. Green, W. H. Slacer, W. C. Earl and S. G. Dorr.

On motion of Dr. Cronyn the paper read by Dr. Mynter at the February meeting was taken from the table for discussion. (See Art. III., Page 377).

DR. CRONYN said that he believed the point intended to be made by the author was the value and advisability of early paracentisis without waiting for absorption or empyema. He believed this to be an important question, and one that demanded serious consideration. He spoke of the difficulty of always recognizing the presence of the fluid, and the dangers which might arise in a mistaken diagnosis. Mr. Waters, of London, had written a paper upon this very subject, his views were that when the pleural cavity was full, and there was considerable constitutional disturbance, with dyspnœa, immediate measures should be taken for the relief of the patient. He warns younger surgeons of the fact that death may suddenly occur during the operation, but does not give the cause for such an occurrence. Before making the operation the surgeon should satisfy himself that absorption can not be promoted by other means.

DR. BARTLETT said that this was a subject which required careful consideration. A careful and accurate diagnosis should be made in the first place, the surgeon should also satisfy himself that his operation was not in danger of aggravating the condition of the patient rather than relieving it. His experience thus far had been chiefly with an expectant treatment, the employment of diuretics and mercurials.

DR. MYNTER said that he had very little to add to what he had said in his paper. He thought that there was but very little danger in the operation, certainly not more than waiting. When the physician waits far the fluid to be carried off by the slow process of absorption, the lung is often so compressed that it cannot expand, as a result of which, we have chronic pneumonia, phthisis, etc. The patient referred to in the paper read at the last meeting, was now perfectly well of his pleurisy.

It is perhaps difficult at times to diagnose these cases with certainty, but even if the diagnosis is not accurate, the introduction of the small clean trocar of the aspirator cannot be productive of much harm should the lung even be punctured.

Dr. Hopkins said that it occurred to him that we should be careful to separate in our minds the operation proposed by Dr. Mynter and the operation for the relief of empyema. Dr. Mynter proposes to operate for the cure of pleuritic effusions in the early days of the disease, and on purely theoretical grounds his operation was plausible.

Dr. Cronyn said that in Dr. Mynter's operation as well as in that for empyema, relief was what was intended at first, cure might perhaps be expected, but he thought the primary object of the operation was to relieve the patient.

Dr. Mynter replied that of course he expected to relieve the patient but that he proposed to operate with an idea of accomplishing a cure.

Dr. Cronyn said that he thought that Dr. Mynter did not intend to recommend this operation unless there was some imperative necessity for the removal of the fluid, as dyspnœa, too great compression of the lungs, etc.

Dr. Mynter said that in the case reported the operation was made as a curative measure, not as a palliative. The condition of the patient was not such as demanded any immediate means for relief. Had it been desirable there was sufficient time for the employment of other means.

Dr. Bartlett said, that in the consideration of this subject, we should, if possible, take into account the question of how many cases recover in ordinary medical practice. We should ask ourselves whether a reasonably large proportion of cases do not recover under ordinary treatment before adopting an operation which must be attended by some danger. He spoke now of its employment as a curative measure in ordinary cases; of course where the accumulation of fluid was rapid or from the large amount present, the patient was in danger from great dyspnœa, it was the only resource left.

Dr. Wyckoff said that he had never seen any very unfavorable results follow an early operation. He had operated on different occasions but always as a palliative measure. In a large number of cases the effusion which came on after the operation was as great or greater than before, and necessitated another operation. He should not operate in cases where the effusion was slight or when the breathing and general condition of the patient was not badly affected.

Dr. Cronyn said that it was always his advice to his younger brethren in the profession never to perform any operation of any magnitude or risk without the co-operation and advice of some older medical man. The slightest operations, even if the diagnosis, etc., were correct, might be followed by serious results and the presence of older and more experienced practitioners would be desirable as a protective measure.

Dr. Wyckoff said that the character of the fluid in cases of the kind under discussion could be very easily ascertained by the use of the ordinary hypodermic syringe.

Dr. Hopkins, spoke of a case of hip-joint disease, of about three months duration, in which the head of the bone was dislocated forward and under Pouparts ligament. The limb is drawn backward and outwards, and fixed in that position. The dislocation was of about three weeks standing.

Dr. Cronyn said that dislocations of the head of the bone in hip-joint diseases were liable to occur in almost any direction. Dr. Harrington brought to the Sisters Hospital, some time since, a little boy with dislocation of the head of the femur upon the dorsum of the illium, as result of disease. The dislocation was reduced, and proper retentive apparatus applied. At last accounts the patient was doing well.

Dr. Brush said that it would seem to him that the cases in which the disease had reached such a point as to render dislocation liable were rare, in which reduction would be productive of any beneficial results. He related a case seen by him, in charge of Dr. J. F. Miner, in which the head of the bone was dislocated upon the pubis.

The disease, a result of injury, had existed for some years. Several sinuses lead down to the bone, the head and neck of which were denuded and roughened. Dr. Miner exsected the head and two inches of the shaft. The operation was undertaken as a last resort, the patient, a young man twenty-four years of age, having tried all other means of treatment before submitting to exsection. After the removal of the head of the femur immediate relief was experienced by the patient, his appetite improved, the discharge, became less, and for a time he improved rapidly. He died at the end of about two weeks from acute peritonitis, probably the result of an abscess opening into the pelvic cavity. The result of exsection of the hip-joint are often surprising. Dr. Sayre, who has had the largest experience of any man in this country if not in the world, has, in over fifty cases, met with a large percentage of success.*

DR. BARTLETT asked in regard to the employment of the extension splints of Sayre and Taylor.

DR. BRUSH, replied that if properly applied at the right period of the disease they prevented further extension of the disease and frequently their use resulted in a cure.

DR. O'BRIEN, in reply to a question as to the prevailing diseases, reported seven cases of small pox only. One in Folsom Street, one on Delevan Avenue beyond Cold Spring and five in the pest house. No special disease seemed to be prevailing.

DR. HOWE spoke in reference to myopia in school children. He referred to the investigations of Donders, Agnew, Williams and others, and said that he proposed to make similar investigations in Buffalo.

DR. LOTHROP moved that the Association endorse Dr. Howe in his investigations, and that he be requested to report the result of his labors to this Association at some future meeting. Carried.

On motion the Association adjourned.

* In a work on Orthopædic Surgery and Diseases of the Joints, just issued by Dr. Sayre, he gives the result of fifty-nine exsections of the hip-joint made by him, as follows:—Whole number of cases fifty-nine, of which thirty-nine are still alive Of the twenty who died eight had recovered from the operatiion some time previous to death, which was caused by some disease foreign to the operation; four of the remaining twelve died of some acute intercurrent disease, tetanus, double pneumonia, dysentery and sun-stroke, leaving but eight who died from the exhaustive effect of hip disease.

Of the thirty-nine still alive, twenty recovered with motion and less than one inch shortening; eight recovered with motion and more than one inch shortening; two recovered with anchylosis, and nine are still under treatment, with every prospect of good results.

